# Supplementary material for: The miR-106b/NR2F2-AS1/PLEKHO2 Axis Regulates Migration and Invasion of Colorectal Cancer through the MAPK Pathway
Source: Int J Mol Sci. 2021 May 30;22(11):5877. doi: 10.3390/ijms22115877 (PMC8198404; doi:10.3390/ijms22115877)
Supplement: Supplementary file 1 [file ijms-22-05877-s001.zip › ijms-1221906-supplementary.pdf]

Supplementary figure 1

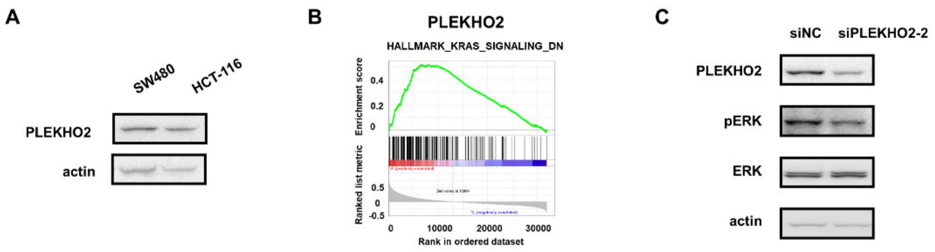

Supplementary figure 1. PLEKHO2 affect the MAPK pathway in CRC. (A) Western blot showed that the protein level of PLEKHO2 is a little higher in SW480 cells than in HCT-116 cells. (B) The GSEA results showed "HALLMARK_KRAS_SIGNALING_DN" was significantly enriched in the high levels of PLEKHO2. (C) Western blot showed that transfection of siPLEKHO2 suppresses the phosphorylation of ERK.
